# Pediatric primary renal lymphoma presenting with multiple masses: A challenging case report and narrative review

**DOI:** 10.1097/MD.0000000000033850

**Published:** 2023-05-17

**Authors:** Xiao He, Min Di, Guan-Nan Wang, Jian-Bo Gao

**Affiliations:** a Ultrasound Department, The First Affiliated Hospital of Zhengzhou University, Zhengzhou, China; b Department of Pathology, The First Affiliated Hospital of Zhengzhou University, Zhengzhou, China; c Department of Radiology, The First Affiliated Hospital of Zhengzhou University, Zhengzhou, China.

**Keywords:** Burkitt lymphoma, case report, needle biopsy, pediatric, primary renal lymphoma

## Abstract

Only 20 cases of pediatric primary renal non-Hodgkin’s lymphoma have been reported since 1995, rare cases and a variety of imaging manifestations have led to difficulties in its diagnosis and treatment.

**Patient concerns:**

Herein, we share in detail a case of primary renal lymphoma (PRL) in a child and summarize the common clinical manifestations, imaging features, and prognostic factors of pediatric PRL by retrospectively analyzing cases reported in the literature. A 2-year-old boy presented to the clinic with a large mass on the right side of his abdomen along with loss of appetite.

**Diagnoses:**

Imaging revealed a large right renal mass, nearly replacing the entire renal tissue, along with numerous small nodules in the left kidney. Given no local adenopathy and metastases, the diagnosis was unclear. A percutaneous renal puncture was performed, which proved the diagnosis of Burkitt’s lymphoma. Since no bone marrow involvement, this child was diagnosed with pediatric PRL.

**Interventions:**

This PRL boy was treated with the NHL-BFM95 protocol and supportive care.

**Outcomes:**

Unfortunately, this boy died of multiple organ failure in the fifth month of treatment.

**Lessons:**

As per literature review, the presentation of pediatric PRL is fatigue, loss of appetite, weight loss, abdominal swelling, or other nonspecific symptoms. Although in 81% of cases it often infiltrates the bilateral kidneys, urine abnormalities caused by pediatric PRL are uncommon. 76.2% of pediatric PRL were boys and 2/3 of all cases presented as diffuse renal enlargement. Those PRL presented as masses could easily be misdiagnosed as WT or other malignancies. Absent of local enlarged lymph node, no necrosis or calcification suggest atypical presentation of renal masses and a percutaneous biopsy is needed in timely establishing the accurate diagnosis for appropriate treatment. Based on our experience, percutaneous renal puncture core biopsy is a safe procedure.

## 1. Introduction

Non-Hodgkin lymphoma (NHL) is the fourth most common diagnosis of pediatric cancer in children. The incidence of NHL increases with age, with a predominance of males. Most pediatric NHL cases are of high grade, such as Burkitt lymphoma (BL), diffuse large B cell lymphoma, lymphoblastic T cell or B cell lymphoma, and anaplastic large cell lymphoma, and have an aggressive clinical behavior in contrast to adults.^[1]^ Timely diagnosis and treatment is extremely important.

Primary renal lymphoma (PRL), one type of NHL, restricting to kidney without other organ infiltration, is extremely rare in children.^[2]^ Given the rarity and greatly overlapped presentations with other malignant renal lesions, pediatric PRL may be easily misdiagnosed.^[3]^ As per literature review, most of the time they were diagnosed post-surgical resection for other clinically diagnosed malignancies. In this paper, the authors share in detail the diagnosis and management of one pediatric bilateral PRL, BL, with a review of the relevant literature on pediatric PRL.

## 2. Case report

**Figure 1.**
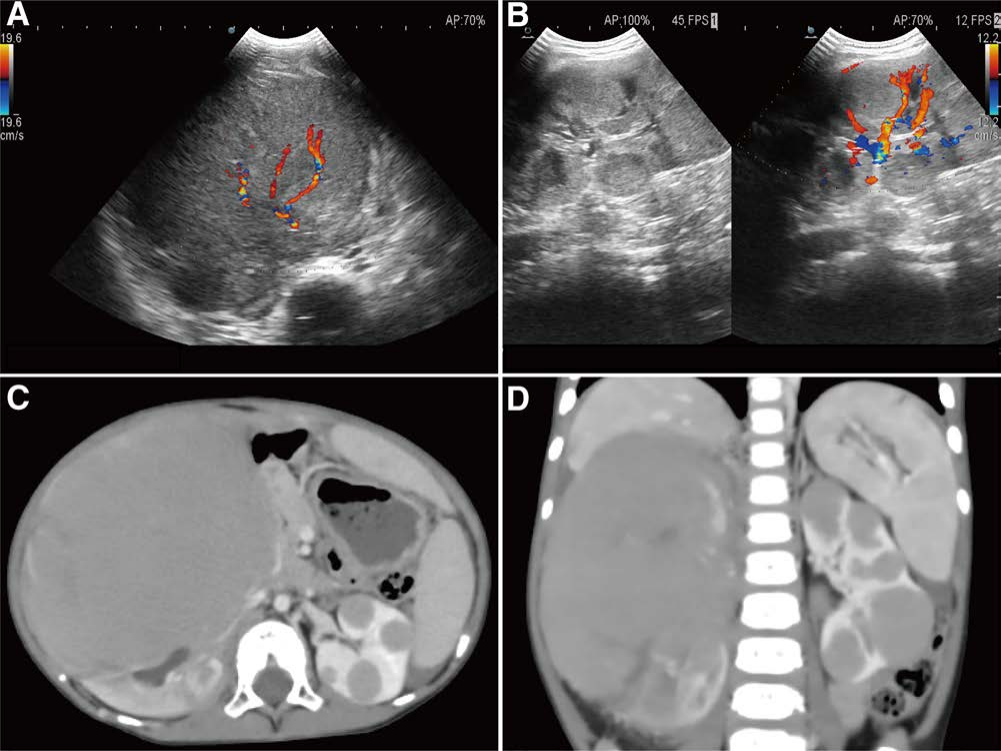
Ultrasonography revealed a solid heterogeneous hypoechoic mass in right abdomen (A); multiple homogeneous isoechoic nodules were seen in the contralateral kidney. (B) Enhanced CT showed the large solid low-density right mass infiltrated the entire right kidney, along with multi well defined nodules in left kidney, without enlarged lymph nodes or signs of metastasis (C and D).

A 2-year-old boy was brought to our clinic with the complaint of an incidental discovery of a large mass in his right abdomen with loss of appetite for 4 days. Physical examination confirmed the mass in the right abdomen, approximately 12 × 11 cm in size. The patient’s family lives in a rural area without any history of familial history of lymphomas or other disease including immunodeficiency-associated diseases or exposure to common carcinogens. An emergency ultrasound exam and laboratory tests was performed. A 131 × 86 × 98 mm solid heterogeneous hypoechoic mass with sparse flow were detected in his right abdominal imaging, with a small portion of normal renal parenchyma around its lower pole (Fig. 1A). Multiple solid homogeneous isoechoic lesions were also identified in his contralateral kidney (Fig. 1B).

The laboratory data revealed elevated cancer antigen 125 (130.00 U/mL, normal range 0.01–35 U/mL), neuron-specific enolase (32.10 ng/mL, normal range 0–25 ng/mL), lactate dehydrogenase (1484 U/L, normal range 109–245 U/L), and α-hydroxybutyrate dehydrogenase (1440 U/L, normal range 72–182 U/L), along with mildly increased white blood cells (14.11 × 10^9^/L, normal range 3.5–9.5 × 10^9^/L) and platelets (372 × 10^9^/L, normal range 125–350 × 10^9^/L), slightly elevated serum uric acid (685 μmol/L, normal range 200–440 μmol/L) and decreased hemoglobin (104 g/L, normal range 130–175 g/L). Serum tests for EB virus, cytomegalovirus, and HIV virus were all negative. Urinalysis revealed positive urine protein. Chest X-ray showed normal findings.

**Figure 2.**
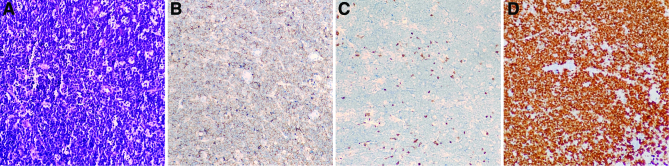
Pathological analysis of biopsy implied diffuse infiltration of medium-sized cells with basophilic cytoplasm, large, circular nuclei, and distinct nuclei (A). Immunohistochemistry showed those cells positive for CD20 (B), negative for CD3 (C), with a positive Ki-67 rate greater than 95% (D). (×200 magnification).

Further, thoraco-abdominal computed tomography (CT) scans were performed. Images of the portal venous phase clearly delineated renal lesions, a 98 × 118 mm low-density mass infiltrating the entire right kidney, along with multiple lesions in the contralateral kidney. The renal capsules remained intact bilaterally (Fig. 1C and D). His clinical diagnosis was favored to be a malignant renal tumor. But given the lack of local adenopathy and signs of metastasis, percutaneous renal mass biopsy was subsequently performed with the consent of his parents for tissue diagnosis. HE staining revealed diffuse infiltration of medium-sized cells with basophilic cytoplasm, large, circular nuclei, and distinct nuclei. A “starry sky” pattern by sparse macrophage phagocytosis of cellular debris was also displayed (Fig. 2A). Immunohistochemical staining illustrated that these cells were positive for CD20, CD10, CD43, bcl-6, negative for CD3, and had a highly robust proliferation rate with a positive Ki-67 staining greater than 95% (Fig. 2B–D). In addition, c-Myc gene translocation was confirmed. Based on these pathological findings, BL was diagnosed. The subsequent bone marrow study revealed no involvement of lymphoma. Bilateral primary renal lymphoma, BL, was established as the final diagnosis.

The patient was treated with NHL-BFM 95 (Non-Hodgkin Lymphoma-Berlin-Frankfurt-Munster 95 Protocol).^[4]^ During chemotherapy, the boy underwent supportive care with NaHCO_3_ to alkalize the urine, allopurinol to reduce uric acid, and granulocyte colony stimulating factor to restore neutrophils. Despite adequate supportive care, the boy died of multiple organ failure in the fifth month of treatment.

## 3. Discussion

The incidence of lymphoma has been on the rise. Most pediatric NHL cases are of high grade with an aggressive clinical behavior in contrast to adults. PRL, a type of non-Hodgkin lymphoma, is confined to the kidney, dose not spread to other organs, and is rare, accounting for 0.7% of extranodal lymphomas.^[5]^ Most PRLs occur in middle-aged and older populations with unilateral renal involvement, with a median age of approximately 72 years.^[6]^ Pediatric PR tends to plague boys, 76.2% of the time (Table 1). Most pediatric PRLs present with fatigue, loss of appetite, weight loss, or abdominal swelling, which are not specific. Clinical manifestations related to the urinary system are rare in pediatric PRL, with only 2 children suffering from hematuria.

**Table 1:** Pediatric primary renal lymphoma since 1995.

Reference	Age/sex	Chief complaint	Site	Imaging findings	Renal function	ARF	LDH	Diagnostic methods	Cell type	Chemotherapeutic protocol	Follow-up
Vujanić et al 1995^[7]^	6/M	Painless hematuria	Left	A mass in the upper pole of the left kidney	Normal	No	NA	Postoperative pathology	DLBCL	NHL 902	Disease free after 2 years
McGuire et al 1996^[8]^	3.5/M	Low grade fever, emesis, anorexia	Bilateral	Enlarged smooth kidneys with multiple bilateral poorly enhancing masses	Impaired	No	487	Surgical biopsy	SNCL	POG protocol 9317	Remission after 4 months
Sieniawska et al 1997^[9]^	11/F	Anorexia, vomiting, general weakness, sleepiness, weight loss	Bilateral	Enlarged kidney without focal mass	Impaired	Yes	NA	Needle biopsy	Burkitt lymphoma	NHL-B 93 protocol	Died during 1st cycle of chemotherapy
Levendoglu-Tugal et al 2002^[10]^	14/M	Headache, flank pain, emesis, weight loss and hypertension	Bilateral	Enlarged kidneys with hemorrhagic foci	Impaired	Yes	622	Needle biopsy	DLBCL	CCG-5942 protocol	Alive after a few months
Sharma et al 2006^[11]^	2.5/M	Abdominal distension, decreased urine output, decreased appetite, nausea, and fatigue.	Bilateral	Bilateral enlarged kidneys without lymphadenopathy	Impaired	Yes	NA	Needle biopsy	T cell NHL	Cyclophosphamide, Vincristine, Prednisolone and L-asparaginase.	Lost in follow-up After 1st cycle of chemotherapy
Becker et al 2007^[12]^	5/M	Hypertension	Bilateral	Bilaterally marked nephromegaly without mass	Impaired	No	6354	Needle biopsy	T-LBL	CCG-1961	Died 2 months after the initial diagnosis
Jindal et al 2009^[13]^	3/M	Abdominal distension, abdominal pain, and fever	Bilateral	Bilateral enlarged kidneys without lymphadenopathy	Impaired	No	NA	Needle biopsy	DLBCL	BFM-90	Died during 5th cycle of chemotherapy
Kumar et al 2010^[14]^	12/M	Right lumber mass	Right	A solid heterogeneous mass arising from right kidney	NA	NA	NA	Needle biopsy	DLBCL	BFM-90 protocol	Disease free after 3 months
Paladugu et al 2010^[15]^	2.5/F	Fever, abdominal distension, vomiting, and decreased urine output	Bilateral	Bilaterally enlarged kidneys without focal masses	Impaired	Yes	2148	Needle biopsy	PTCL	Vincristine, prednisone, Daunomycin, and L-asparaginase	Disease free at 20 months
Dash et al 2011^[16]^	7/F	Fever, joint pain, anemia, and distended abdomen.	Bilateral	Bilateral diffused renal enlargement	Normal	No	NA	Needle biopsy	DLBCL	CHOP	NA
Hayakawa et al 2013^[17]^	12/F	Gross hematuria	Right	An enhanced lesion in the superior pole of the right kidney	Normal	No	NA	Postoperative pathology	DLBCL	Hyper-CVAD	Remain alive and disease free 3years after treatment
Dhull et al 2015^[18]^	8/F	Fever, joint pain, anemia, and distended abdomen	Bilateral	Bilateral renal enlargement with homogenous cortical echogenicity	Impaired	Yes	NA	Needle biopsy	DLBCL	R-CHOP	Remission after 1 year
Butani et al 2017^[19]^	12/M	Fatigue, anemia	Bilateral	Bilaterally enlarged kidneys with no enhancing nodules	Impaired	Yes	NA	Needle biopsy	DLBCL	CHOP	Remains in remission over 4 years
Coca et al 2017^[20]^	10/M	Flank mass	Left	An enlarged left kidney with loss of internal morphological architecture	Normal	No	NA	Postoperative pathology	B cell NHL	NHL-BFM 95	Died at 14 months after diagnosis
South 2018^[21]^	4/M	Fatigue, weight loss	Bilateral	Bilateral nephromegaly with mild loss of corticomedullary differentiation	Impaired	Yes	NA	Needle biopsy	DLBCL	R-CHOP	Remains in remission over 4 years
Aydin Köker et al 2019^[22]^	2.5/M	Abdominal distension	Bilateral	Diffuse bilateral enlarged kidneys with no normal renal parenchyma	Normal	No	1376	Needle biopsy	T-LBL	NHL-BFM 95	Died on the eighth day of chemotherapy
Bruce et al 2020^[23]^	12/M	Headache, persistent nausea with vomiting	Bilateral	Bilaterally enlarged kidneys with left mild hydronephrosis	Impaired	No	285	Needle biopsy	DLBCL	R-CHOP	Remains in remission
Lei et al 2020^[24]^	13/M	Fatigue, anorexia, arthralgia, and weight loss.	Bilateral	Bilateral renal symmetrical enlargement	Impaired	No	367	Needle biopsy	T-LBL	hyper-CVAD	Died at 17 months after diagnosis
Yang et al 2020^[25]^	8/M	Abdominal pain and vomiting	Bilateral	A large irregular mass in the right kidney and multiple nodules in the left kidney.	NA	NA	NA	Needle biopsy	B-LBL	Hyper-CVAD	NA
Ninh et al 2021^[26]^	4/M	Oliguria, weakness, anorexia, vomiting, and abdominal pain	Bilateral	Bilateral enlarged kidney with multiple poorly defined hypo enhancing areas.	Impaired	Yes	540	Needle biopsy	Burkitt lymphoma	EPOCH-R	Remission after 3 cycles
This case report	2/M	Incidental discovery of a large mass in right abdomen	Bilateral	Multiple well-defined masses	Impaired	Yes	1484	Needle biopsy	Burkitt lymphoma	NHL-BFM95	Died in the fifth month of treatment

ARF = acute renal failure, B-LBL = B lymphoblastic lymphoma, CHOP = cyclophosphamide, doxorubicin, vincristine, and prednisone, DLBCL = diffuse large B cell lymphoma, hyper-CVAD = fractionated cyclophosphamide, vincristine, doxorubicin, and dexamethasone, LDH = lactate dehydrogenase, NA = not available, NHL = non-Hodgkin’s lymphoma, PTCL = peripheral T-cell lymphoma, R-CHOP = cyclophosphamide, doxorubicin, vincristine, prednisone and rituximab, SNCL = small noncleaved cell lymphoma, T-LBL = T lymphoblastic lymphoma.

Pediatric PRL commonly involves bilateral, and is often manifested as diffuse enlarged kidney (Table 1). Given the lack of lymphoid tissue in the renal parenchyma, the pathogenesis of PRL may be attributed to the infiltration of lymphocytes into the renal capsule and parenchyma mainly during chronic inflammation. Lymphoma cells grow and proliferate through percolation in the renal interstitium via structures that act as scaffolds, such as glomeruli, renal tubules, collecting tubules and vascular beds. The infiltrated parenchyma appears swollen but with normal structure and profile, resulting in diffuse renal enlargement. Some lymphoma cells grow into a mass compressing the surrounding papillae. This growth form of lymphoma is not uncommon, accounting for about one-third of all patients (Table 1). In addition, a 5% PRL presents with tumor thrombosis in the renal vein or inferior vena cava.^[2]^ The protean presentation of PRLs increase the difficulty of clinical diagnosis.

Due to its convenience and radiation-free nature, the ultrasound is an initial test for scanning pediatric abdominal masses. The ultrasonographic appearance of most PRLs shows diffuse enlargement of the kidney with unclear corticomedullary boundaries. A small fraction of RPL exhibit multiple masses, with 10% to 25% being single, and some of these masses have such low echogenicity that they can be easily be mistaken for cysts.^[27]^ As a further delineation of renal tumors, magnetic resonance imaging (MRI) is recommended as the best imaging modality. The PRL is largely isointense on the T1-weighted images, slightly hypointense on T2-weighted images, and hyperintense on diffusion-weighted images.^[28]^ The volumetric T2 sequence can finely delineate renal lesions anatomically and provides high-resolution vascular details of the lesion. Diffusion-weighted images helps to provide useful information in the detection of small renal lesions and lymphovascular invasion and to distinguish malignant from benign via apparent diffusion coefficient values.^[29]^ But the use of MRI in children has been hampered by the long duration of its scans, which require sedation or general anesthesia. Considering it more cost-effective, CT replaced MRI as the most common test for the detailed assessment of pediatric renal tumors. Considering CT as a standard assessment of pulmonary metastasis, thoracic-abdominal CT can both reduce scan time and the radiation burden, and portal venous phase is sufficient for renal tumor delineation and vessel identification. PRL typically presents as a heterogeneous enlarged kidney, whereas focal PRL may present as a homogeneous internal hypodense lesion with well-defined margins, given that the lesion is less enhanced than the surroundings.^[30]^

However, for pediatric PRL presenting with masses, the information obtained through imaging is insufficient to distinguish it from Wilms tumor (WT), which accounts for approximately 90% of pediatric renal cancers and has a significantly higher incidence in children younger than 4 years of age.^[3]^ Based on the age and imaging features of those multiple well-defined solid renal masses, WT were often diagnosed. The unnecessary, potentially harmful, surgeries were performed in the literature. In our case, there were features with were inconsistent with WTs including bilateral kidney involvement, no signs of necrosis, adenopathy or metastases prompted us for further investigation with percutaneous renal mass biopsy.

Percutaneous renal mass biopsy is not routinely recommended for children younger than 10 years of age, due to the risks associated with renal biopsy, including bleeding and reduced availability of diagnostic tissue. It is reported unlikely to change clinical strategies.^[31,32]^ But when non-WT, especially NHL, is a possible differential diagnosis, biopsy should be considered. Percutaneous puncture biopsy accounted for 76.2% of the diagnoses of PRL in children (Table 1). With the available immunohistochemistry and flow cytometry, the likelihood of accurate tumor diagnosis and typing has significantly increased. In our case, an ultrasound-guided percutaneous biopsy of the right renal tumor was performed without any complications, by which a diagnosis of renal lymphoma was established, and an unnecessary nephrectomy or other inappropriate chemotherapy was avoided.^[27]^

BL presented as pediatric PRL is very rare, although it is the most common pathological subtype of pediatric non-Hodgkin’s lymphoma (NHL), accounts for about 30% to 50% of NHL cases, with boys having a considerably higher incidence.^[33]^ The median age of BL was 8 years old, with more than 1/3 of the cases in children aged 5 to 9 years. BL has 3 subtypes based on clinical and genetic characteristics. Sporadic BL, occurring primarily in the abdomen, is the dominant type in China. Because BL progresses rapidly and is likely the fastest-growing tumor in humans, early intervention significantly improves the prognosis.^[34]^

Several short-course, high-dose, multi-drug combination chemotherapy strategies are recommended for BL. High-dose methotrexate is a key component of the treatment regimen. SFOP-LMB and NHL-BFM are 2 classic treatment strategies for pediatric B-cell lymphoma and achieve an overall disease-free survival rate of 80% to 90%.^[4,35]^ Several studies have shown that the addition of rituximab can help improve NHL outcomes at various risk levels.^[36,37]^ However, given the myelotoxic effects, high cost, and drug resistance in the relapse stage,^[38]^ this PRL child in the Stage III of the St. Jude grading system was treated with the classic NHL-BFM 95 chemotherapy strategy without rituximab.

The prognosis for pediatric PRL remains poor, with a fatality rate of 1/3. Elevated serum lactate dehydrogenase (LDH) and uncommon pathological types are significantly correlated with poor prognosis (Table 1). LDH plays an influential role in evaluating the prognosis of melanomas, germ cell tumors, non-Hodgkin’s lymphoma, and acute leukemia reaction. Both abnormally vigorous anaerobic metabolism in tumor cells and tissue necrosis increase serum LDH levels, suggesting a heavier tumor burden.^[39]^ In our case, serum lactate dehydrogenase (LDH) levels higher than 1000 U/L indicated a poor prognosis.^[40]^ Despite supportive care during chemotherapy, the child still died from septic shock, multiple organ dysfunction and respiratory failure 5 months after diagnosis.

In summary, percutaneous biopsies of pediatric renal tumors with atypical images are safe and necessary to successfully diagnose PRL and to avoid unnecessary nephrectomies. To facilitate accurate management of pediatric renal masses, it is necessary to convert clinical and imaging data into a simple scoring system with quantitative thresholds to increase the efficiency of renal mass biopsy.

## 4. Conclusion

Diagnosing pediatric PRLs is challenging, especially when it presents as a renal mass. Imaging data plays a role in describing lesions, and portal venous phase-enhanced CT is sufficient for detailed delineation. However, imaging of PRLs has low diagnostic accuracy due to multiple manifestations. A percutaneous renal mass biopsy is necessary in the treatment of atypical pediatric renal lesions. The benefit-to-risk ratio of percutaneous needle biopsies in children increased with the use of immunocytochemistry and flow cytometry. A pathological diagnosis of PRL can avoid incorrect interventions. Supportive care is essential in the treatment of pediatric PRL to prevent tumor lysis syndrome, which can help improve survival for this rare malignancy.

## Author contributions

**Resources:** Xiao He, Min Di.

**Validation:** Guan-Nan Wang.

**Writing – original draft:** Xiao He, Min Di.

**Writing – review & editing:** Min Di, Jian-Bo Gao.
